# Hepatocellular carcinoma cells downregulate NADH:Ubiquinone Oxidoreductase Subunit B3 to maintain reactive oxygen species homeostasis

**DOI:** 10.1097/HC9.0000000000000395

**Published:** 2024-03-04

**Authors:** Zhendong Zhang, Qianwei Zhao, Zexuan Wang, Fang Xu, Yixian Liu, Yaoyu Guo, Chenglong Li, Ting Liu, Ying Zhao, Xiaolei Tang, Jintao Zhang

**Affiliations:** 1 Henan Institute of Medical and Pharmaceutical Sciences, Zhengzhou University, Zhengzhou, China; 2 BGI College, Zhengzhou University, Zhengzhou, China; 3 Henan Key Medical Laboratory of Tumor Molecular Biomarkers, Zhengzhou University, Zhengzhou, China; 4 School of Basic Medical Sciences, Academy of Medical Sciences, Zhengzhou University, Zhengzhou, China; 5 Department of Veterinary Biomedical Sciences, College of Veterinary Medicine, Long Island University, Brookville, New York, USA; 6 Department of Medicine, Division of Regenerative Medicine, School of Medicine, Loma Linda University, Loma Linda, California, USA; 7 Department of Basic Science, School of Medicine, Loma Linda University, Loma Linda, California, USA; 8 Henan Key Laboratory of Tumor Epidemiology, Zhengzhou University, Zhengzhou, China; 9 State Key Laboratory of Esophageal Cancer Prevention & Treatment, Zhengzhou University, Zhengzhou, China

## Abstract

**Background:**

HCC is a leading cause of cancer-related death. The role of reactive oxygen species (ROS) in HCC remains elusive. Since a primary ROS source is the mitochondrial electron transport chain complex Ι and the NADH:ubiquinone Oxidoreductase Subunit B3 (NDUFB3), a complex I subunit, is critical for complex I assembly and regulates the associated ROS production, we hypothesize that some HCCs progress by hijacking NDUFB3 to maintain ROS homeostasis.

**Methods:**

NDUFB3 in human HCC lines was either knocked down or overexpressed. The cells were then analyzed in vitro for proliferation, migration, invasiveness, colony formation, complex I activity, ROS production, oxygen consumption, apoptosis, and cell cycle. In addition, the in vivo growth of the cells was evaluated in nude mice. Moreover, the role of ROS in the NDUFB3-mediated changes in the HCC lines was determined using cellular and mitochondrion-targeted ROS scavengers.

**Results:**

HCC tissues showed reduced NDUFB3 protein expression compared to adjacent healthy tissues. In addition, NDUFB3 knockdown promoted, while its overexpression suppressed, HCC cells’ growth, migration, and invasiveness. Moreover, NDUFB3 knockdown significantly decreased, whereas its overexpression increased complex I activity. Further studies revealed that NDUFB3 overexpression elevated mitochondrial ROS production, causing cell apoptosis, as manifested by the enhanced expressions of proapoptotic molecules and the suppressed expression of the antiapoptotic molecule B cell lymphoma 2. Finally, our data demonstrated that the apoptosis was due to the activation of the c-Jun N-terminal kinase (JNK) signaling pathway and cell cycle arrest at G0/G1 phase.

**Conclusions:**

Because ROS plays essential roles in many biological processes, such as aging and cancers, our findings suggest that NDFUB3 can be targeted for treating HCC and other human diseases.

## INTRODUCTION

Liver cancer is a leading cause of cancer-related death and one of the most prevalent cancers worldwide. The majority of primary liver cancer is HCC. Numerous new targeted therapies and immunotherapies are under clinical trials to manage the disease,^1^ significantly improving response rate and prolonging survival time. However, except for those suitable for curative therapies, most HCCs gradually progress under current treatments. Therefore, HCC therapy is still an unmet medical need. Consequently, it is imperative to understand the mechanisms underlying HCC progression better to discover novel therapeutic targets.

Concerning the mechanisms of HCC progression, reactive oxygen species (ROS) are being actively studied.^2^ Early analysis showed that human cancer cells produced more ROS than normal cells.^3^ Because ROS can damage DNA, proteins, and lipids, it was thought that elevated ROS levels could increase genomic instability to promote tumorigenesis.^4^ Recent data further suggest ROS modulates numerous signaling pathways in cancer cells, heightening cancer progression, and metastasis.^5^ The role of ROS in cancer development and progression led to the hypothesis that antioxidants might be potential cancer treatments.^6,7^ However, multiple randomized, placebo-controlled clinical trials failed to substantiate this concept.^8,9^ Paradoxically, some preclinical studies showed that antioxidants promoted cancer progression and metastasis.^10^

In addition to antioxidants, prooxidants have been proposed as potential cancer therapies.^11^ The rationale is that because of high levels of ROS, cancer cells’ antioxidant system has reached its functional peak, and further increases in ROS levels can lead to cell death.^12,13^ These previous findings underscore the necessity of understanding the role of ROS in cancer development.

Cellular ROS are produced primarily by mitochondria and NADPH oxidases.^14^ In mitochondria, previous data suggest that the electron transport chain (ETC) complex I is the primary site of ROS production.^15,16^ Mammalian complex I is the largest and least understood in the ETC. It has 45 subunits. The core subunits include 7 mitochondrion-encoded and 7 nucleus-encoded subunits, which are highly conserved across species. All the complex I subunits have been isolated^17^; however, the functions of individual subunits remain elusive.^18^ Although complex I is the main contributor of ROS that participates in many signaling pathways under physiological conditions,^19^ the exact site of ROS production within complex I is not fully understood.^16,19^

The regulation of ROS production in complex I also remains obscure. Available data indicate that protonmotive force (Δp), NADH/NAD ratio, CoQH_2_ (reduced coenzyme Q)/coenzyme Q ratio, and local O_2_ concentration determine the rate of ROS production.^15,20^ In addition, recent findings reveal that complex I subunit NDUFB3 (NADH:ubiquinone oxidoreductase subunit B3) regulates ROS production,^21^ and NDUFB3 downregulation is associated with thyroid cancer progression.^22^

NDUFB3 is located in the membrane arm’s distal proton pumping module (Pd) of complex I.^23,24^ It is methylated,^25^ and it has been proposed that the methylation by the assembly factor C20orf7 is a critical step in early complex I assembly.^26^ This notion was further supported by a previous report that discovered a mutation in the C20orf7 that led to only 30%–40% complex I assembly, diminished complex I activity, and caused Leigh syndrome.^27^ In addition to the C20orf7, a recent report showed that methyltransferase METTL9 also mediated NDUFB3 methylation, and its enzymatic activity was required for optimal complex I function.^28^ Therefore, available data support that NDFUB3 is essential for normal complex I activity^29,30^ and regulates ROS production. In this study, we hypothesize that some HCCs progress by hijacking and downregulating NDUFB3 to maintain ROS homeostasis.

## METHODS

### Animals

Female BALB/c nude mice were purchased from the Vital River Laboratories and housed in a standard barrier environment at the Zhengzhou University animal facility. The animals were allowed for acclimation about a week before experimentation. The Animal Care and Use Committee at Zhengzhou University approved this study.

### Bioinformatic analysis

The Cancer Genome Atlas (TCGA) RNA-seq data were analyzed using the The University of Alabama at Birmingham Cancer data analysis Portal platform (http://ualcan.path.uab.edu). Protein expression data were extracted from the Clinical Proteomics Tumor Analysis Consortium (CPTAC) database.

### Sample collection from patients with HCC

Twenty-four pairs of tumors and matched adjacent nontumorous liver tissues were obtained from patients with HCC in the First Affiliated Hospital of Zhengzhou University (Supplemental Table S1, http://links.lww.com/HC9/A826). The patients did not receive any medical treatment before surgery. The paired nontumor tissues were collected at least 2 cm from the primary tumor tissues. The tissue specimens were washed with PBS and stored in liquid nitrogen. Written consent was given in writing by all subjects. All research was conducted in accordance with the Declarations of Helsinki and Istanbul. This study was approved by the Research Ethics Committee of Zhengzhou University.

### Cell lines

The following HCC cell lines were used in this study: HepG2 (Genechem Co.), Huh7 (Genechem Co), and SNU-449 (Chinese Academy of Sciences). The following immortalized hepatocyte cell line was used as control: QSG-7701 (Biowing Company).

### Generation of stable NDUFB3-overexpressing Huh7 cell line

NDUFB3 overexpression was achieved by transducing cells with the lentivirus expressing the NDUFB3 gene with a Flag&His tag (LV-NDUFB3, Hunan Fenghui Biotech). Hence, the exogenously overexpressed NDUFB3 has a molecular weight of 12 kDa as compared to the 11 kDa endogenous NDUFB3. The primer sequences used to obtain the NDUFB3 gene are shown in Supplemental Table S2, http://links.lww.com/HC9/A827.

Briefly, the cells (2×10^5^ cells/well) were added into a 6-well plate and incubated at 37°C and 5% CO_2_. After reaching 70% confluence (typically 24 hours later), the cells were transduced with the LV-NDUFB3 (the MOI for Huh7 cells was 5). Positively transduced cells were selected by puromycin (2 µg/mL).

### RNA isolation and reverse transcription-quantitative PCR

According to the manufacturer’s instructions, total RNA was extracted from cells using TRIzol reagent (Invitrogen; Thermo Fisher Scientific, Inc.). RNA (2 µg) was reverse transcribed into cDNA using the PrimeScript RT Reagent kit. Reverse transcription-quantitative PCR was performed using SYBR Green (Roche Diagnostics, Mannheim, Germany). The thermocycling conditions were as follows: 95°C for 10 minutes; 35 cycles of 95°C for 60 seconds (denaturation), 56°C for 60 seconds (annealing), and 72°C for 2 minutes (extension), followed by 72°C for 6 minutes. Glyceraldehyde-3-phosphate dehydrogenase served as an internal control. Relative gene expression was quantified using the 2^−ΔΔCT^ method. Primers used in this study are shown in Supplemental Table S2, http://links.lww.com/HC9/A827.

### Western blot analysis

The tissues or cells were lysed using radioimmunoprecipitation assay buffer (Solarbio) containing phenylmethylsulfonyl fluoride (Solarbio) at 4°C for 30 minutes and centrifuged at 13,000 rpm for 15 minutes at 4°C. The supernatants were collected and measured for protein concentrations using the bicinchoninic acid protein assay (Thermo Fisher Scientific). Proteins (50 µg) were boiled and separated by SDS-PAGE gel. Next, the proteins were transferred to a 0.45 μm Polyvinylidene fluoride membrane (Merck Millipore), and the membrane was blocked for 1.5 hours in nonfat powdered milk (BBI Life Sciences) and then incubated with a primary antibody at 4°C overnight. The primary antibodies used in this study were as follows: anti-NDUFB3 (BBI Life Sciences), anti-CDK4 (BBI Life Sciences), anti-Bcl-2-associated X protein (Wanleibio), anti-c-Jun N-terminal kinase (JNK) (Wanleibio), anti-phospho-JNK (Wanleibio), anti-cyclin D1 (ProteinTech Group), anti-p21 (ProteinTech Group), anti-cytochrome C (ProteinTech Group), anti-B cell lymphoma 2 (BCL2) (ProteinTech Group), anti-cleaved Poly (ADP-ribose) polymerase (PARP) (Cell Signaling Technology), anti-cleaved caspase 9 (Cell Signaling Technology, polyclonal antibodies, detect a 37 kDa band; Wanleibio, polyclonal antibodies, detect a 17 kDa band), anti-cleaved caspase 3 (Cell Signaling Technology), and anti-glyceraldehyde-3-phosphate dehydrogenase (Cell Signaling Technology).

Following the primary antibody incubation, the membrane was washed 3 times with Tris-buffered saline with Tween 20 and incubated with a secondary antibody (HRP-conjugated Goat Anti-Rabbit IgG, diluted in Tris-buffered saline with Tween 20) for 2 hours. Subsequently, the membrane was washed 3 times with Tris-buffered saline with Tween 20. Protein bands were detected using the ECL chemiluminescence system.

### Cell proliferation assay

Cell proliferation was determined using the cell counting Ki-8. Briefly, 2500 cells/100 μL/well were seeded in a 96-well plate. At 24, 48, 72, and 96 hours, the cells were treated with the kit reagents for 2 hours. Subsequently, the absorbance value was evaluated at the wavelength of 450 nm by using an automated microplate reader (Bio-Rad).

### Colony formation assay

In short, about 3000 cells/2 mL/well of cells in DMEM culture medium were inoculated in a 6-well plate and cultured for 2 weeks. Then, the cells were washed with PBS, fixed with 4% paraformaldehyde for 20 minutes, and stained with 0.5% crystal violet for 30 minutes at room temperature. Colonies were photographed and counted with an optical microscope (Olympus Corp).

### Invasiveness and migration assay

For the invasion assay, matrigel (BD Biosciences) was melted overnight at 4°C. The melted matrigel was diluted in DMEM/high glucose medium (HyClone) (Matrigel: medium=1:6). Then, 30 µL of the diluted matrigel was added into the upper chamber of a transwell plate with 8-µm pore sizes (Corning). The plate was incubated at 37°C and 5% CO_2_ for 4 hours (the matrigel solidified during this period). For the migration assay, there was no need to add matrigel into the upper chamber.

For both the invasion and migration assays, the DMEM/high glucose medium (HyClone) was added to the plate to moisten the membrane twice, 15 minutes each time. Then, 600 μL of the DMEM/high glucose medium containing 10% fetal bovine serum was added into lower chambers. In addition, 8×10^4^ cells/well in 600 µL of the DMEM/high glucose medium (without serum) were added into the up chambers. The plate was then incubated for 48 hours.

Following the incubation, the upper chambers were washed with PBS, and the cells on the membrane surface were removed gently using cotton swabs. Subsequently, the membranes were fixed in absolute methanol and stained with 0.1% crystal violet solution. Cells in the membranes were counted under a microscope.

### Flow cytometry analysis of apoptosis

Apoptosis was analyzed using the Annexin V-Allophycocyanin/7-Amino Actinomycin D apoptosis Detection Kit (KeyGEN). Briefly, cells in the logarithmic phase were digested with trypsin without EDTA, pelleted at 1200 rpm for 4 minutes, and washed twice with cold PBS. Then, 100 μL Binding Buffer was used to suspend the cells. Subsequently, 5 μL of the 7-Amino Actinomycin D and Annexin V-Allophycocyanin was added and mixed. The cells were incubated at room temperature in the dark for 10 minutes. The cells were then added with 400 µL binding buffer, mixed evenly, and analyzed on ACEA NovoCyte3130 within 1 hour.

### Cell cycle analysis

The cells were seeded into a 6-well plate. When the number of cells reached 80%, the cells were collected and washed twice with cold PBS. Then, the cells were fixed with 70% ethanol at 4°C overnight. Next, the cells were stained with PI and RNase A for 30 minutes at room temperature in darkness, and DNA content was detected by CytoFLEX S flow cytometer (Beckman Coulter).

### Wound healing assay

Three parallel lines were marked in each well’s center on the outside bottom of a 6-well culture plate. Then, the cells (5×10^5^ cells/2 mL/well) were inoculated into the plate and cultured at 37°C and 5% CO_2_. After the cells reached 80% confluence, the medium was discarded. Wounds were drawn along the 3 parallel lines using a 200 μL pipette, and the scratched space was cleaned with PBS so no residual cells were present. The plate was cultured at 37°C and 5% CO_2_ and photographed at 0 and 48 hours using a CKX53 microscope (Olympus). The wound healing rate was calculated according to the following formula: wound healing rate=[(scratch width at 0 hours − scratch width at 48 hours)/(scratch width at 0 hours)]×100%.

### Detection of intracellular ROS production by dihydroethidium probe

Briefly, the cells were inoculated into a 24-well plate (8×10^3^ cells/well). When the cell density reached 80%, the old medium was discarded, and the cells were washed twice gently with PBS. Then, the cells were added with dihydroethidium (10 μM), and cells were incubated at 37°C for 10 minutes in the darkness. Later, cells were washed twice with PBS, and the fluorescence intensity representing ROS content was detected using the Eclipse TS100 microscope (Nikon) and analyzed by ImageJ software.

### Determination of mitochondrial complex Ι (NADH ubiquinone oxidoreductase) activity

According to the manufacturer’s instructions, the enzymatic activity of mitochondrial complex Ι was detected using the Micro Mitochondrial Complex Ι Activity Assay Kit (Solarbio). Briefly, 5×10^6^ cells were suspended with 1 mL solution Ι and homogenized for 30 seconds. The cells were centrifuged (600 g) at 4°C for 10 minutes. Then, the supernatant was collected and centrifuged (11,000*g*) at 4°C for 15 minutes. The pellets containing mitochondria were collected. Solution I (200 µL) and solution II (200 µL) were subsequently added, and the solution was sonicated 15 times (200 W, 5 seconds per time). After that, 10 µL sample, 154 µL reaction buffer I, 20 µL working buffer, and 16 µL reaction buffer IV were added to a preheated 96-well plate in order, and the absorbance at 340 nm was measured as A1. Then, the plate was incubated at 37°C for 1 minute and measured with the absorbance at 340 nm as A2. The complex Ι activity was calculated according to the formula: mitoch ondrial complex I activity (U/mg prot)=[ΔA ×V_sum_ ÷ (ε×d)×10^9^] ÷ (V_sample_ ×Cpr) ÷ T. ΔA: A1-A2; V_sum_: Total volume of the reaction buffer (2×10^−4^ L); ε: NADH molar absorptivity (6.22 ×10^3^ L/mol/cm); d: 0.6 cm; V_sample_: The volume of sample (0.01 mL); T: reaction time (1 minute); Cpr: the concentration of protein (mg/mL).

### Measurement of oxygen consumption rate

The oxygen consumption rate was measured using the BBoxiProbeTM R01 (BestBio) on the CLARIOstar ACU instrument (BMG LABTECH). The main components of this kit were an oxygen-sensitive fluorescein probe solution and mineral oil. Briefly, Huh7 cells (60,000 cells/100 μL/well) were seeded in a 96-well plate and cultured at 37°C and 5% CO_2_. The following day, the culture plate’s old medium was discarded and replaced with 150 μL/well of preheated fresh medium. Then, 10 μL/well of the probe solution was added to each well, and the plate was sealed with 2 drops of preheated mineral oil. The plate was then placed in the prewarmed CLARIOstar ACU instrument, and the oxygen consumption rates in the cells were measured for 120 minutes.

### Tumor growth in nude mice

Ten 4-week-old female BALB/c nude mice were divided into 2 groups and marked with ear labels to distinguish them. 1×10^7^ control or NDUFB3-overexpressing Huh7 cells in 100 μL were inoculated subcutaneously under the right axilla. When tumors were visible (typically 7 days after inoculation), a Vernier caliper was used to measure tumor sizes every other day. Tumor volumes were calculated using the following formula: length×width^2^×0.5.

In addition, the mice were imaged in a small animal live imager 1 and 3 weeks after the cell injection. Immediately following the second time of imaging, the mice were euthanized. Tumors were isolated, weighed, and photographed.

### Immunohistochemistry staining

In brief, the excised tumor tissues were fixed in 4% formaldehyde, followed by embedding in paraffin. The tissue sections were deparaffinized with xylene and rehydrated in graded alcohols, followed by incubation in 3% hydrogen peroxide for 30 minutes. The sections were heated at 98°C for 10 minutes in EDTA buffer for antigen retrieval, blocked with 3% Bovine serum albumin for 30 minutes, incubated with anti-NDUFB3 antibody overnight at 4°C, and then with secondary antibody at room temperature.

### Generation of stable NDUFB3 knockdown HepG2 cell line

NDUFB3 expression in HepG2 cells was knocked down using small hairpin RNA (shRNA). Briefly, shRNA targeting NDUFB3 and a negative control shRNA were purchased from Genechem Co., Ltd. The sequences of the shRNA are shown below:


Control-shRNA(sense):5′-TTCTCCGAACGTGTCACGT-3′;

NDUFB3-shRNA(sense):5′-CCGCAATGAAGCTTGGAGATA-3′;

Twenty-four hours before transduction, the cells (2×10^5^ cells/well) were added into a 6-well plate and incubated at 37°C and 5% CO_2_. After reaching 70% confluence, the cells were transduced with the control-shRNA or the NDUFB3-shRNA. Positively transduced cells were selected by puromycin (2 μg/mL).

### Statistical analysis

All statistical analyses were performed with GraphPad Prism 7.0 software. Comparison between 2 or more groups was done using the two-tailed Student *t* test or one-way ANOVA. Data were presented as mean± SD. A *p*-value<0.05 was considered statistically significant.

## RESULTS

### NDUFB3 is downregulated in tumor tissues of patients with HCC

**FIGURE 1:**
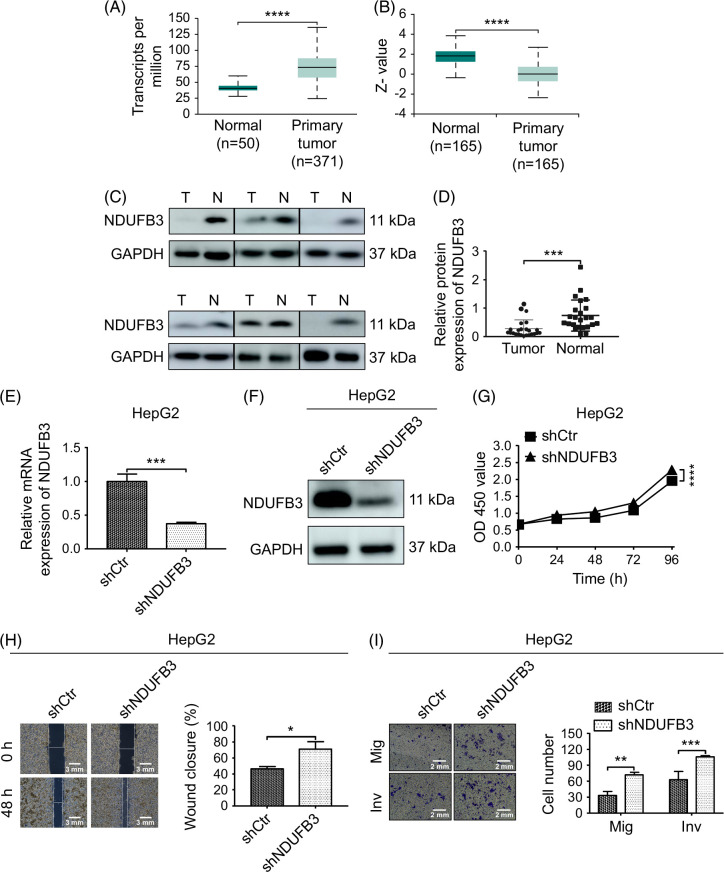
NDUFB3 knockdown promotes HCC cells’ growth, migration, and invasiveness. (A) The TCGA database was used to analyze the mRNA expression of NDUFB3 in HCC (primary tumor) and normal tissues (normal). (B) NDUFB3 protein expression levels in HCC and normal tissues were analyzed using the CPTAC database. (C and D) Twenty-four paired HCC (T) and matched normal liver tissues (N) were obtained from the First Affiliated Hospital of Zhengzhou University. NDUFB3 protein expression levels were measured by western blot. Representative gel images (C) and cumulative data (D) are shown. Data show mRNA (E) and protein (F) expression of NDUFB3 in HepG2 cells transduced with control-shRNA (shCtr) or NDUFB3-shRNA (shNDUFB3) lentiviral vector. (G) The growth of the shCtr-transduced or shNDUFB3-transduced HepG2 cells was monitored in real-time using CCK-8 analysis. (H) The wound healing assay was used to determine the migrating ability of the shCtr-transduced or shNDUFB3-transduced HepG2 cells. Representative images (left) and cumulative data (right) are shown. (I) The migration (Mig) and invasiveness (Inv) of the shCtr-transduced and shNDUFB3-transduced HepG2 cells were evaluated using the Transwell analysis. Representative images (left) and cumulative data (right) are shown. *p<0.05, **p<0.01, ***p<0.001, and ****p<0.0001. Abbreviations: GAPDH, glyceraldehyde-3-phosphate dehydrogenase; NDUFB3, NADH:ubiquinone oxidoreductase subunit B3; TCGA, The Cancer Genome Atlas.

To understand the role of NDUFB3 in HCC, we analyzed its mRNA and protein expressions in the TCGA and CPTAC databases, respectively. Although NDUFB3 mRNA expression was substantially increased (Figure 1A), its protein expression was significantly downregulated (Figure 1B). Since prognosis data were unavailable in the CPTAC database, we used the TCGA database to examine the relationship between NDUFB3 mRNA expression levels and survival time. The data showed that NDUFB3 mRNA expression levels were inversely correlated with the survival time (Supplemental Figure S1A, http://links.lww.com/HC9/A825). In addition, high NDUFB3 mRNA expression levels were associated with significantly lower survival probability (Supplemental Figure S1B, http://links.lww.com/HC9/A825). Moreover, higher disease stages (stage II, III, and IV) displayed substantially higher NDUFB3 mRNA expression levels compared to the lower disease stage (stage I) (Supplemental Figure S1C, http://links.lww.com/HC9/A825).

Interestingly, available data in the TCGA and CPTAC databases showed that the mRNA and protein expressions of many other complex I subunits displayed a similar pattern (Supplemental Figure S2A, B, http://links.lww.com/HC9/A825). Because previous reports suggest that NDUFB3 is critical for the complex I assembly, regulates associated ROS production, and is associated with cancer development,^21,22,26^ we performed further investigation on NDUFB3.

To confirm the observations in the CPTAC databases, we investigated the NDUFB3 protein expression in HCC and matched normal liver tissues from 24 patients treated at the First Affiliated Hospital of Zhengzhou University. This analysis corroborated the CPTAC database findings, showing that NDUFB3 protein expression was significantly reduced in HCC tissues compared to normal liver tissues (Figure 1C, D).

In addition to the clinical HCC tissue samples, we compared NDUFB3 mRNA and protein expressions in commonly used HCC cell lines (Huh7, SNU-449, and HepG2 cells) with normal immortalized hepatocyte (QSG-7701 cells). Reverse transcription-quantitative PCR analysis showed that the NDUFB3 mRNA expression was significantly downregulated in the Huh7 and SNU-449 cells, whereas it remained at a similar level in the HepG2 cells (Supplemental Figure S3A, http://links.lww.com/HC9/A825). In addition, western blot examination revealed that NDUFB3 protein expression in the Huh7 and SNU-449 cells was substantially decreased, while it was not significantly altered in the HepG2 cells (Supplemental Figure S3B, C, http://links.lww.com/HC9/A825). Hence, the NDUFB3 protein downregulation in some HCCs is consistent throughout all the samples. In the following studies, we knocked down NDUFB3 expression in the HepG2 cells and overexpressed it in the Huh7 cells to capture NDUFB3’s role in HCC development.

### NDUFB3 knockdown promotes HCC cells’ growth, migration, and invasiveness

On the basis of the above analyses, we posited that NDUFB3 downregulation was a survival strategy that some HCCs developed. Among the 3 HCC cell lines analyzed, the mRNA and protein expressions of NDFUB3 in the HepG2 cells were similar to the normal hepatocyte (QSG-7701 cells). Hence, we asked how knocking down NDUFB3 expression might affect the functions of the HepG2 cells. We successfully knocked down NDUFB3 expression at mRNA (Figure 1E) and protein (Figure 1F) levels. Cell Counting kit-8 (CCK-8) analysis showed that NDUFB3 knockdown increased HepG2 cell proliferation (Figure 1G). Additionally, wound healing and transwell migration examinations revealed that NDUFB3 knockdown promoted HepG2 cell migration (Figure 1H, I). Furthermore, the transwell invasiveness evaluation demonstrated that NDUFB3 knockdown enhanced NDUFB3 invasiveness (Figure 1I). Hence, our data suggest that NDUFB3 downregulation is a viable strategy that HCCs can adopt for their benefit.

### NDUFB3 overexpression suppresses HCC cells’ growth, migration, and invasiveness

**FIGURE 2:**
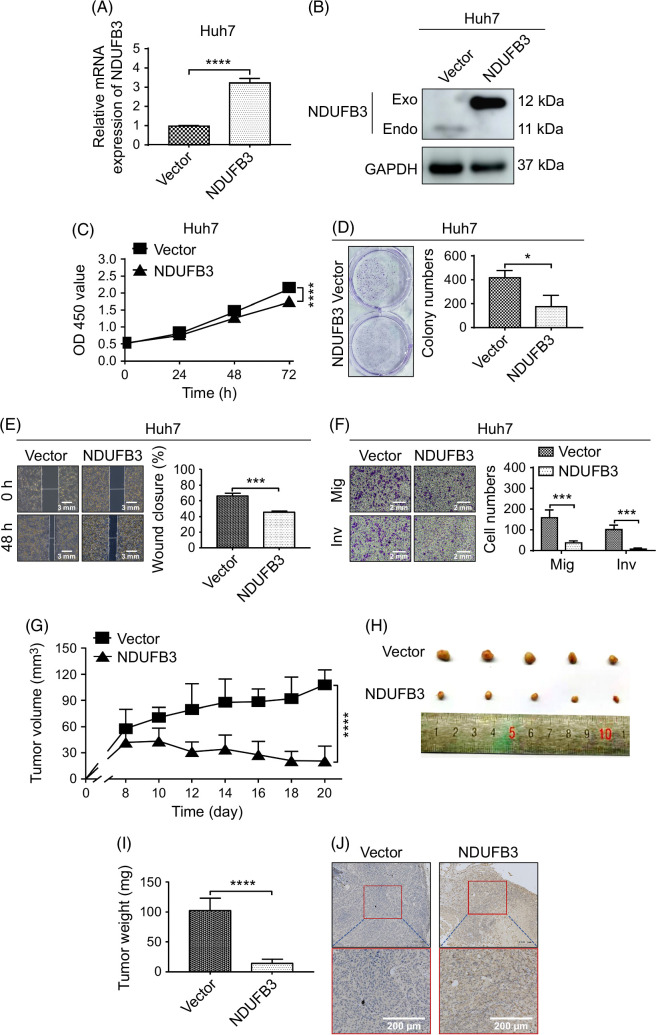
NDUFB3 overexpression suppresses HCC cells’ growth, Mig, and Inv. Reverse transcription-quantitative PCR (A) and western blot (B) were used to analyze the mRNA and protein expression of NDUFB3 in Huh7 cells that were transduced with either the control (vector) or NDUFB3-overexpressing (NDUFB3) lentiviral vector. (C) The effects of NDUFB3 overexpression on the proliferation of Huh7 cells were examined by CCK-8 analysis. (D) The effect of NDUFB3 overexpression on the colony formation of Huh7 cells. Representative images (left) and cumulative data (right) are shown. (E) The effect of NDUFB3 overexpression on the wound healing of Huh7 cells. Representative images (left) and cumulative data (right) are shown. (F) The effect of NDUFB3 overexpression on the Mig and Inv of Huh7 cells. Representative images (left) and cumulative data (right) are shown. (G) Huh7 cells transduced with either the control or the NDUFB3-overexpressing vector were subcutaneously injected into nude mice, and the tumor volumes were measured every other day. Data show the tumor volumes within the 20-day observation period. (H) On day 20, tumors were isolated from the nude mice. Images show the sizes of individual tumors. (I) Data show the average weights of the isolated tumors. (J) NDUFB3 expression in isolated tumor tissues was analyzed by immunohistochemistry. Data show representative images of the staining. *p<0.05, ***p<0.001, and ****p<0.0001. Abbreviations: Endo, endogenous; Exo, exogenous; GAPDH, glyceraldehyde-3-phosphate dehydrogenase; Inv, invasiveness; Mig, migration; NDUFB3, NADH:ubiquinone oxidoreductase subunit B3.

To determine NDUFB3’s role in HCC further, we overexpressed NDUFB3 in Huh7 cells. The overexpression was confirmed with reverse transcription-quantitative PCR (Figure 2A) and western blot (Figure 2B).

CCK-8 analysis showed that NDUFB3 overexpression significantly reduced the proliferation of the Huh7 cells (Figure 2C). In addition, NDUFB3 overexpression also decreased the colony-forming capability of Huh7 cells (Figure 2D). Moreover, the wound healing and transwell migration assays demonstrated that the migration ability of Huh7 cells (Figure 2E, F) was significantly compromised after NDUFB3 overexpression. Consistently, the transwell invasiveness assay revealed that NDUFB3 overexpression substantially suppressed the invasiveness of Huh7 cells (Figure 2F).

In addition to the ex vivo experiments, we investigated the role of NDUFB3 in HCC cell growth in vivo. Specifically, control or NDUFB3-overexpressing Huh7 cells were subcutaneously injected into the right axilla of nude mice. One week later, the tumor volume was measured every 2 days. Results showed that NDUFB3 overexpression significantly decreased the tumor volumes (Figure 2G). In addition, the mice were imaged in a small animal live imager 1 and 3 weeks after the cell injection. The imaging data revealed that the tumor sizes in the mice injected with the NDUFB3-overexpressing Huh7 cells were much smaller than those with the control cells (Supplemental Figure S4, http://links.lww.com/HC9/A825). Immediately following the second imaging, the mice were sacrificed, and the tumors were collected for analysis. Our data uncovered that NDUFB3 overexpression reduced tumor sizes (Figure 2H) and weights (Figure 2I). The overexpression of NDUFB3 in tumor tissues was confirmed by immunohistochemistry (Figure 2J).

### NDUFB3 knockdown significantly decreases, whereas its overexpression increases complex I activity

**FIGURE 3:**
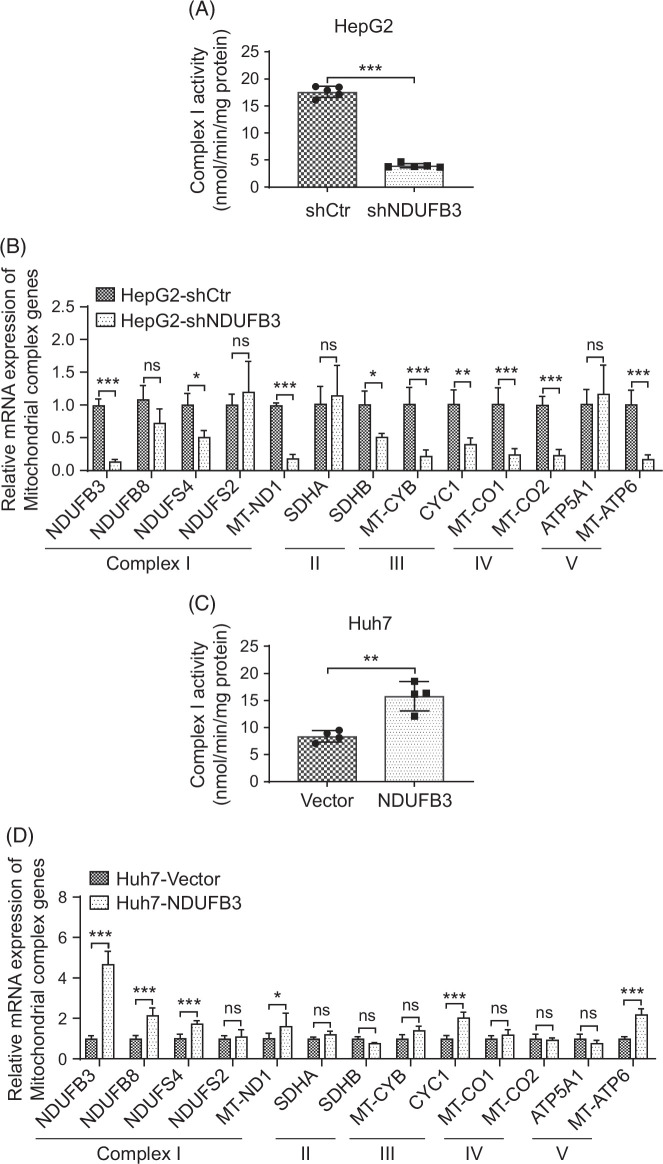
NDUFB3 knockdown significantly decreases, whereas its overexpression increases complex I activity. (A, B) NDUFB3 in HepG2 cells was knocked down, as described in Figure 1. Complex I activity in the control (shCtr) and NDUFB3 knockdown (shNDUFB3) HepG2 cells were determined as described in Methods (A). In addition, the mRNA expressions of the selected subunits of complex I, II, III, IV, and V were quantified by reverse transcription-quantitative PCR (B). (C, D) NDUFB3 in Huh7 cells was overexpressed, as described in Figure 2. Complex I activity (C) in the control (vector) and NDUFB3-overexpressing (NDUFB3) Huh7 cells were measured. Besides, the mRNA expressions of the selected subunits of complex I, II, III, IV, and V in the corresponding Huh7 cells were also quantified. *p<0.05, **p<0.01, and ***p<0.001. Abbreviations: NDUFB3, NADH:ubiquinone oxidoreductase subunit B3; ns, not significant; shCtr, shRNA, small hairpin RNA.

Because it had been proposed that NDUFB3 was a critical step in early complex I assembly^25–28^ and regulated associated ROS production,^21^ we reasoned that NDUFB3 downregulation helped maintain ROS balance in HCC cells by keeping complex I activity at appropriate levels, thereby promoting tumor progression. Hence, we asked how NDUFB3 knockdown and overexpression affected the complex I activity. We first measured the complex I activity in HepG2 cells before and after NDUFB3 knockdown. Our data showed that NDUFB3 knockdown significantly decreased the complex I activity (Figure 3A). In addition, the NDUFB3 knockdown also reduced the expressions of other subunits in complex I, II, III, IV, and V (Figure 3B).

We then overexpressed NDUFB3 in Huh7 cells. Our data demonstrated that NDUFB3 overexpression significantly increased the complex I activity in Huh7 cells (Figure 3C). Similarly, NDUFB3 overexpression elevated the expressions of other subunits in complexes I, II, III, IV, and V (Figure 3D).

### NDUFB3 overexpression enhances ROS production and apoptosis in HCC cells

**FIGURE 4:**
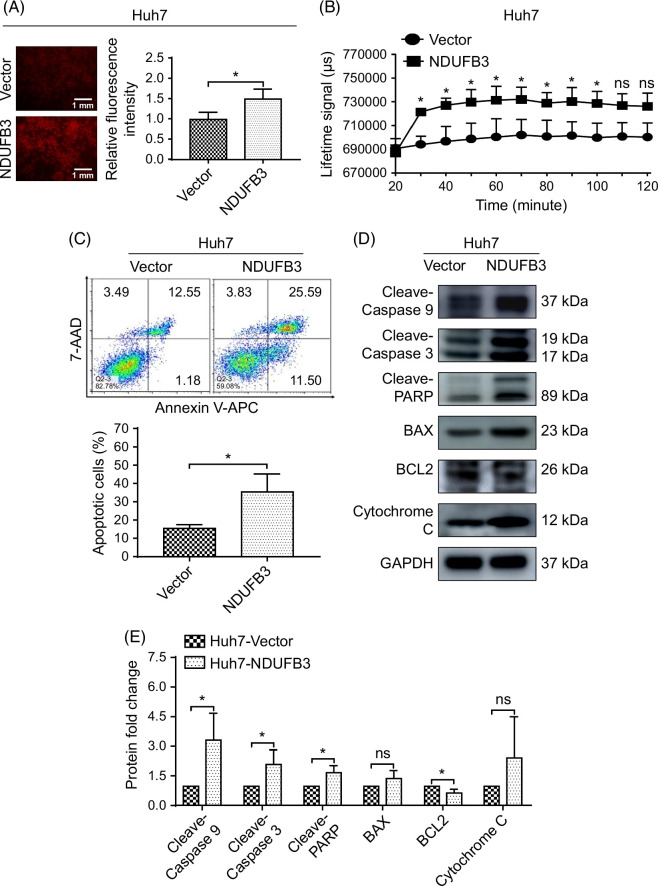
NDUFB3 overexpression enhances the production of ROS and apoptosis in HCC cells. (A) ROS in control (vector) and NDUFB3-overexpressing (NDUFB3) Huh7 cells were examined. Representative images (left) and cumulative data (right) are shown. (B) Oxygen consumption rate in the control and NDUFB3-overexpressing Huh7 cells is shown. (C) Apoptosis of the control and NDUFB3-overexpressing Huh7 cells was determined using flow cytometry analysis. Representative plots (up) and cumulative data (down) are shown. (D and E) Apoptosis-associated molecules, including cleaved caspase-9 (detected using the Cell Signaling Technology antibody), cleaved caspase-3, cleaved PARP, BAX, BCL2, and cytochrome C, were examined using western blot analysis. Representative gel images (D) and cumulative data (E) are shown. *p<0.05. Abbreviations: AAD, Amino Actinomycin D; BAX, Bcl-2-associated X protein; BCL2, B cell lymphoma 2; PARP, Poly (ADP-ribose) polymerase; NDUFB3, NADH:ubiquinone oxidoreductase subunit B3; ns, not significant; V-APC, V-Allophycocyan.

Because ETC complex Ι is a major contributor to cellular ROS, too high ROS levels could exceed HCC cells’ antioxidant capacity and induce apoptosis. For this reason, we determined ROS production in HCC cells. Our data demonstrated that NDUFB3 overexpression significantly elevated ROS levels in Huh7 cells (Figure 4A). In addition, we also observed increased oxygen consumption in these cells (Figure 4B).

In line with the increased cellular ROS levels, flow cytometry showed that the proportion of apoptotic cells was significantly enhanced in NDUFB3-overexpressing Huh7 cells compared to the control cells (Figure 4C). In addition, we examined the expression levels of the critical factors involved in cell apoptosis. The data revealed that, in the NDUFB3-overexpressing Huh7 cells compared to the control cells, the expressions of the proapoptotic proteins, including cleaved caspase 9, cleaved caspase 3, cleaved PARP, Bcl-2-associated X protein, and cytochrome C were increased. In contrast, the antiapoptotic protein BCL2 was significantly downregulated (Figure 4D, E).

### NDUFB3 overexpression induces cell cycle arrest and the activation of JNK signaling pathway in HCC cells

**FIGURE 5:**
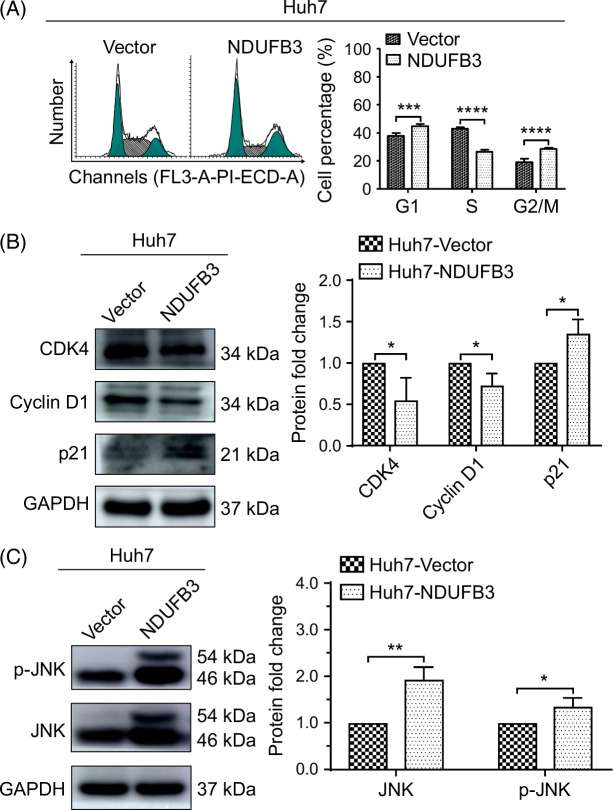
NDUFB3 overexpression induces cell cycle arrest and the activation of JNK signaling pathway in HCC cells. (A) Cell cycle analysis of the control (vector) and NDUFB3-overexpressing (NDUFB3) Huh7 cells. Representative FACS plots (left) and cumulative data (right) are shown. (B) Western blot analysis of the molecules that regulate G1/S stage, including CDK4, cyclin D1, and p21 in the control and NDUFB3-overexpressing Huh7 cells. Representative images (left) and cumulative data (right) are shown. (C) Western blot analysis for the expression levels of JNK and p-JNK. Representative gel images (left) and cumulative data (right) are shown. *p<0.05, **p<0.01, ***p<0.001, and ****p<0.0001. Abbreviations: GAPDH, glyceraldehyde-3-phosphate dehydrogenase; JNK, c-Jun N-terminal kinase; NDUFB3, NADH:ubiquinone oxidoreductase subunit B3.

High ROS levels have been shown to trigger cellular apoptosis through at least 2 mechanisms, i.e., cell cycle arrest^31^ and the activation of JNK signaling pathway.^32,33^ Our analysis of cell cycle distribution in Huh7 cells with and without NDUFB3 overexpression showed that the number of the cells in G0/G1 phase increased, while that in the S phase significantly decreased in the NDUFB3-overexpressed Huh7 cells (Figure 5A). The data suggested a cell cycle arrest at G0/G1 phase. Because cyclin D1 and CDK4 functions are required for cell cycle progression from the G0/G1 phase to the S phase, and p21 inhibits their functions, we examined the expression of cyclin D1, CDK4, and p21. Our data showed that NDUFB3 overexpression enhanced the protein expression of p21 but reduced the protein expression of CDK4 and cyclin D1 (Figure 5B), supporting the NDUFB3-mediated G0/G1 arrest.

We then measured the expression levels of JNK and phosphorylated JNK (p-JNK). Our data demonstrated significantly increased levels of JNK and p-JNK in Huh7 cells following NDUFB3 overexpression (Figure 5C).

### Inhibition of ROS accumulation reverses the effects of NDUFB3 overexpression

**FIGURE 6:**
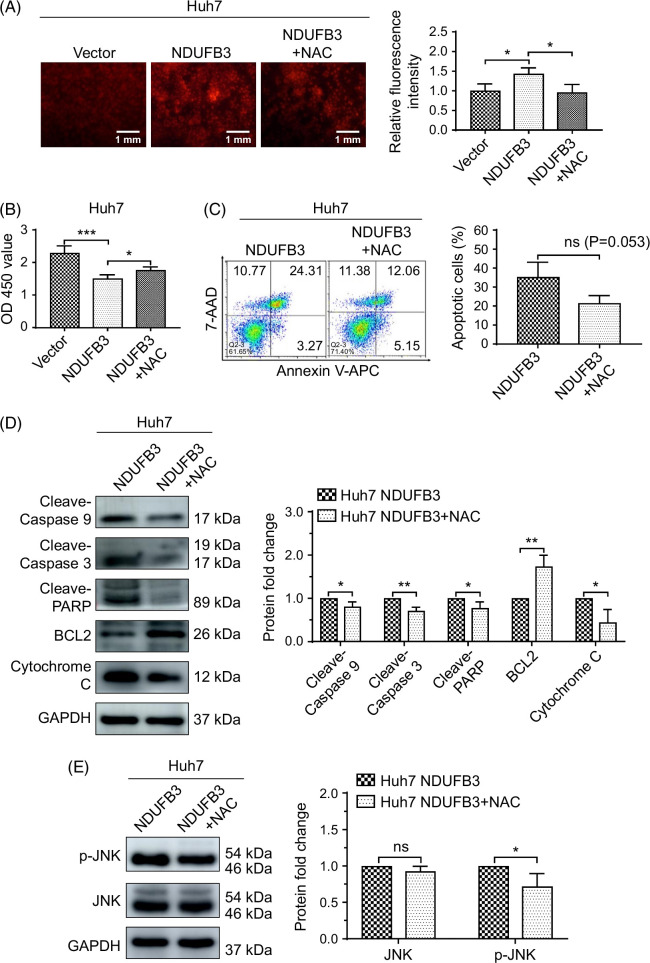
Inhibition of cellular ROS production reverses the effects of NDUFB3 overexpression. Huh7 cells were transduced with either the Vector or NDUFB3 lentiviral vector. In addition, a portion of the NDUFB3-transduced cells was also treated with NAC (10 nM), a cellular reactive oxygen species scavenger, for 24 hours for the following analyses: (A) reactive oxygen species production: representative images (left) and cumulative data (right) are shown; (B) proliferation using cell counting Kit-8 analysis; (C) apoptosis: representative FACS plots (left) and cumulative data (right) are shown; (D) the expression of the molecules associated with apoptosis by western blot: representative gel images (left) and cumulative data (right) are shown (the cleaved-Caspase 9 was detected using the Wanleibio antibody); (E) the expression of JNK and p-JNK by western blot: representative gel images (left) and cumulative data (right). *p<0.05, **p<0.01, and ***p<0.01. Abbreviations: AAD, Amino Actinomycin D; BCL2, B cell lymphoma 2; GAPDH, glyceraldehyde-3-phosphate dehydrogenase; JNK, c-Jun N-terminal kinase; NAC, N-acetyl-l-cysteine; NDUFB3, NADH:ubiquinone oxidoreductase subunit B3; ns, not significant; PARP, Poly (ADP-ribose) polymerase; V-APC, V-Allophycocyanin.

Next, we asked whether removing excess ROS could reverse the proapoptotic effect of NDUFB3 overexpression. We used N-acetyl-l-cysteine (NAC), a widely used cellular ROS scavenger,^34^ to answer this question. We confirmed that NDUFB3 overexpression increased ROS levels in Huh7 cells and that NAC treatment significantly reduced ROS levels in the NDUFB3-overexpressing Huh7 cells (Figure 6A). Consistently, the NAC treatment enhanced the proliferation, migration, and invasiveness of the NDUFB3-overexpressing Huh7 cells (Figure 6B, Supplemental Figure S5, http://links.lww.com/HC9/A825), which was accompanied by reduced apoptosis (Figure 6C). In addition, western blot analysis showed that the NAC treatment significantly decreased the expression levels of proapoptotic molecules, including cleaved caspase 9, cleaved caspase 3, cleaved PARP, and cytochrome C, and increased the expression level of antiapoptotic BCL2 in NDUFB3-overexpressing Huh7 cells (Figure 6D). The above analyses aligned with the finding that NAC treatment significantly reduced the expression of p-JNK (Figure 6E).

**FIGURE 7:**
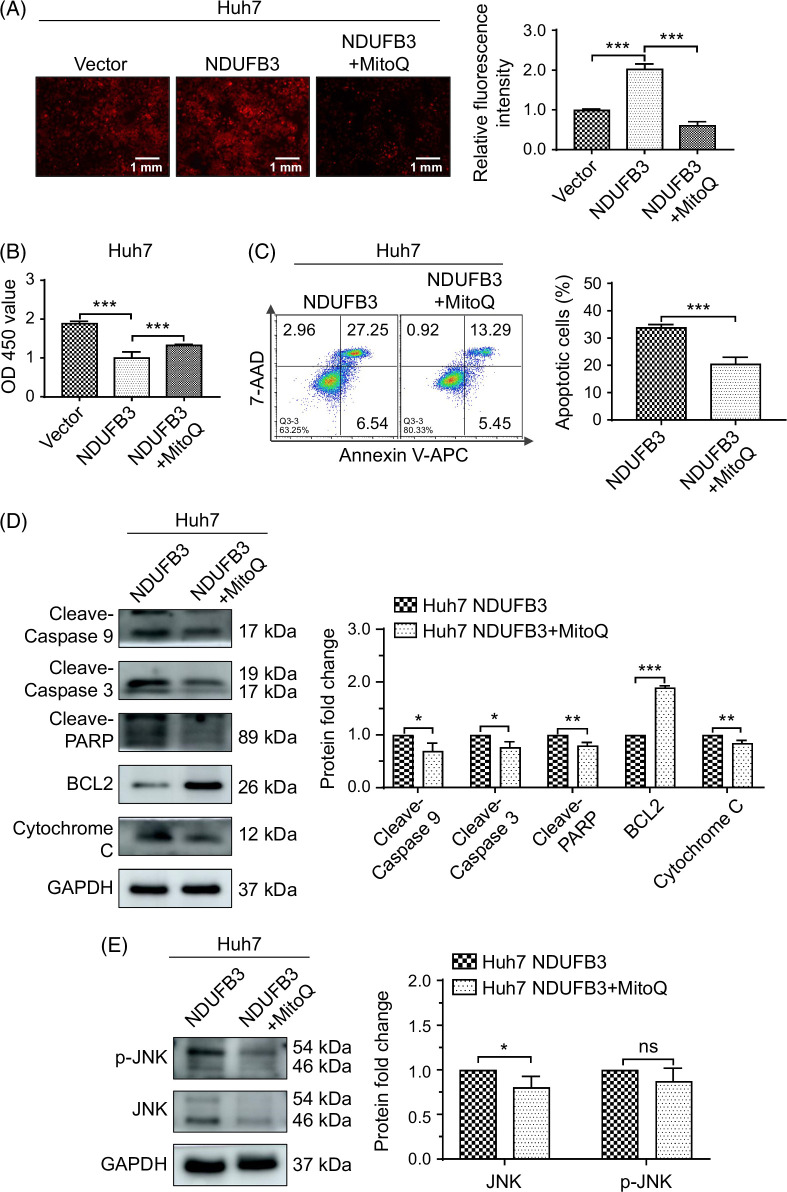
Inhibition of mitochondrial reactive oxygen species production reverses the effects of NDUFB3 overexpression. Huh7 cells were transduced with either the vector or NDUFB3 lentiviral vector. In addition, a portion of the NDUFB3-transduced cells was also treated with mitoquinone mesylate (MitoQ, 5 μM) (MCE MedchemExpress, CAS No: 845959-50-4, USA), a mitochondrially targeted reactive oxygen species scavenger, for 30 hours for the following analyses: (A) reactive oxygen species production: representative images (left) and cumulative data (right) are shown; (B) proliferation using CCK-8 analysis; (C) apoptosis: representative FACS plots (left) and cumulative data (right) are shown; (D) the expression of the molecules associated with apoptosis by western blot: representative gel images (left) and cumulative data (right) are shown (the cleaved-Caspase 9 was detected using the Wanleibio antibody); (E) the expression of JNK and p-JNK by western blot: representative gel images (left) and cumulative data (right). *p<0.05, **p<0.01, and ***p<0.01. Abbreviations: AAD, Amino Actinomycin D; BCL2, B cell lymphoma 2; GAPDH, glyceraldehyde-3-phosphate dehydrogenase; JNK, c-Jun N-terminal kinase; NDUFB3, NADH:ubiquinone oxidoreductase subunit B3; ns, not significant; PARP, Poly (ADP-ribose) polymerase; V-APC, V-Allophycocyanin.

One may argue that NAC inhibits all cellular ROS, and NDUFB3 overexpression only increases mitochondrial ROS production. To further the role of mitochondrial ROS, we used mitoquinone mesylate, a mitochondrion-targeted ROS scavenger. Our data using mitoquinone mesylate were similar to NAC (Figure 7).

## DISCUSSION

This study found that the NDUFB3 was downregulated in some HCCs. NDUFB3’s knockdown promoted, while its overexpression suppressed HCC cells’ growth, migration, and invasiveness. In addition, NDUFB3 knockdown significantly decreased, whereas its overexpression increased complex I activity. Further studies revealed that NDUFB3 overexpression increased mitochondrial ROS production, causing cell apoptosis through activating JNK signaling pathway and cell cycle arrest. Our data suggest that some HCCs progress by hijacking and downregulating NDUFB3 expression to maintain ROS homeostasis. Hence, NDUFB3 is a potential target for prooxidant therapy of HCC.

NDUFB3 is a subunit of ETC complex I. Our discovery of NDUFB3’s role in maintaining ROS balance in HCC supports the previously proposed NDUFB3 function in determining normal complex I activity and regulating ROS production.^25–27^ In addition to the NDUFB3, many other ETC complex I subunits also display reduced protein expressions in HCC (Supplemental Figure S2, http://links.lww.com/HC9/A825). Since we observed that NDUFB3 knockdown and overexpression were associated with downregulation and upregulation, respectively, of other subunits in complex I, II, III, IV, and V (Figure 3), the downregulation of other complex I subunit protein expressions in HCC can be secondary to the NDUFB3 downregulation. The mechanisms by which NDUFB3 regulates other ETC complexes warrant further investigation.

A recent study also shows the importance of downregulating complex I-derived ROS in HCC progress. This study showed that an increased expression of the complex I inhibitor NDUFA4L2 led to reduced ROS production and rendered HCC cells resistant to sorafenib, a first-line medication for advanced HCC.^35^

In addition to its role in cancers, ROS also impacts many other biological processes.^36^ A recent report suggests that complex Ι suppression in oocytes represents an evolutionarily conserved strategy that allows longevity because of low cellular ROS production.^37^ Other studies also show that complex Ι inhibition attenuates myocardial ischemia/reperfusion injury by suppressing ROS production.^38^ Therefore, balancing ROS production is crucial to maintaining normal physiological homeostasis.

One detrimental effect of ROS is the induction of apoptosis. ROS may induce apoptosis through at least 2 mechanisms. First, it has been shown that ROS induces the phosphorylation and activation of proteins involved in the mitogen-activated protein kinase signaling pathways, including JNK, the extracellular signaling-regulated kinase, and p38.^32,33,39,40^ Among the mitogen-activated protein kinase signaling pathways, both JNK and p38 signaling pathways play an essential role in mitochondrial dysfunction and oxidative stress and the initiation of ROS-induced apoptosis.^40–42^ Consistent with these previous findings, in this study, we show that the expression levels of JNK and p-JNK are elevated in NDUFB3-overexpressing HCC cells (Figure 5). In addition, the elevated p-JNK expression is due to a high ROS production because treatment with NAC (a cellular ROS scavenger) (Figure 6) and mitoquinone mesylate (a mitochondrion-targeted ROS scavenger) (Figure 7) abolish both JNK pathway activation and mitochondrial apoptosis in NDUFB3-overexpressed HCC cells.

Excess ROS also triggers cell cycle arrest, which can cause apoptosis.^43^ Cell cycle progression primarily depends on cyclins and cyclin-dependent kinases (CDKs).^44^ Our data show that NDUFB3 overexpression leads to cell cycle arrest at G0/G1 stage (Figure 5). At the start of G1, cyclin D1 forms a complex with CDK4/6, which is required for cell cycle progression to the S phase. Our data demonstrate that NDUFB3 overexpression downregulates the expression of cyclin D1 and CDK4 (Figure 5). In addition, p21 is a CDK inhibitor. Interestingly, NDUFB3 overexpression increases p21 expression (Figure 5). The effects of NDUFB3 on the cell cycle are caused mainly by increasing mitochondrial ROS production, considering that NDUFB3 is a subunit of ETC complex Ι.

**FIGURE 8:**
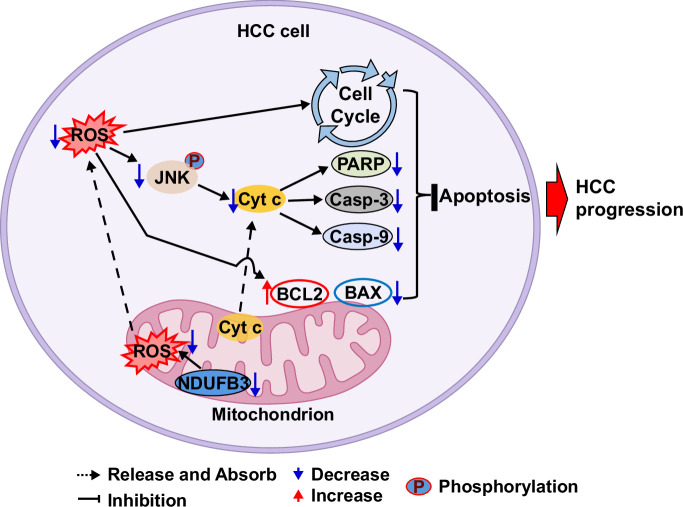
A model of HCC progression via NDUFB3 downregulation. HCC cells downregulate their NDUFB3 expression to counter the elevation of ROS levels due to enhanced metabolic needs. This counter mechanism promotes HCC progression by inducing cell cycle progression and inhibiting cell apoptosis (i.e., suppressing the expression of proapoptotic molecules such as cytochrome c, caspase 3, caspase 9, PARP, and BAX but enhancing the expression of antiapoptotic molecules such as BCL2). Abbreviations: BAX, Bcl-2-associated X protein; BCL2, B cell lymphoma 2; JNK, c-Jun N-terminal kinase; NDUFB3, NADH:ubiquinone oxidoreductase subunit B3; PARP, Poly (ADP-ribose) polymerase; ROS, reactive oxygen species.

Based on our findings, we propose a model of HCC progression via NDUFB3 downregulation (Figure 8). In this model, HCC cells downregulate their NDUFB3 expression to counter the elevation of ROS levels as a result of enhanced metabolic needs. This counter mechanism promotes HCC progression by inducing cell cycle progression and inhibiting cell apoptosis (i.e., suppressing the expression of proapoptotic molecules such as caspase 9, caspase 3, PARP, Bcl-2-associated X protein, and cytochrome c but enhancing the expression of antiapoptotic molecules such as BCL2).

In conclusion, our study suggests that NDUFB3 can be targeted for regulating mitochondrial ROS production. However, our study has several limitations. First, it is not known whether NDUFB3 methylation and complex I assembly are altered in HCC cells, which can help further understand the role of NDUFB3 in regulating ROS production and energy generation in HCC cells. Second, despite the exciting prognosis data in the TCGA gene expression database, no prognosis data in the CPTAC protein expression database and our collected hospital samples are available. Third, other Complex I subunits have also been implicated in regulating ROS and energy generation.^35,45,46^ Future studies are warranted to determine the interaction between NDUFB3 and other complex I subunits.

## Supplementary Material
