# Supplementary material for: Hepatocellular carcinoma cells downregulate NADH:Ubiquinone Oxidoreductase Subunit B3 to maintain reactive oxygen species homeostasis
Source: Hepatol Commun. 2024 Mar 4;8(3):e0395. doi: 10.1097/HC9.0000000000000395 (PMC10914236; doi:10.1097/HC9.0000000000000395)
Supplement: Supplementary file 1 [file hc9-8-e0395-s001.doc]

**Hepatocellular Carcinoma Cells Downregulate NADH:ubiquinone Oxidoreductase Subunit B3 to Maintain Reactive Oxygen Species Homeostasis**

Zhendong Zhang^1,2☆^, Qianwei Zhao^1,4☆^, Zexuan Wang^1,2☆^, Fang Xu^1,4^, Yixian Liu^1,4^, Yaoyu Guo^2^, Chenglong Li^3^, Ting Liu^2^, Ying Zhao^1^, Xiaolei Tang^6.7*^, Jintao Zhang^1,4,5*^

^1^Henan Institute of Medical and Pharmaceutical Sciences, Zhengzhou University, Zhengzhou, China

^2^BGI College, Zhengzhou University, Zhengzhou, China.

^3^School of Basic Medical Sciences, Academy of Medical Sciences, Zhengzhou University, Zhengzhou, China.

^4^Henan Key Medical Laboratory of Tumor Molecular Biomarkers, Zhengzhou University, Zhengzhou, China.

^5^Henan Key Laboratory of Tumor Epidemiology and State Key Laboratory of Esophageal Cancer Prevention & Treatment, Zhengzhou University, Zhengzhou, China.

^6^Department of Veterinary Biomedical Sciences, College of Veterinary Medicine, Long Island University, Brookville, New York, USA.

^7^Division of Regenerative Medicine, Department of Medicine, Department of Basic Science, School of Medicine, Loma Linda University, Loma Linda, CA, USA.

^☆^These authors contributed equally.

*Corresponding authors:

Jintao Zhang: Henan Institute of Medical and Pharmaceutical Sciences, Zhengzhou University, Zhengzhou, China. Email: jtzhang@zzu.edu.cn.

Xiaolei Tang: Department of Veterinary Biomedical Sciences, College of Veterinary Medicine, Long Island University, Brookville, New York, USA. Division of Regenerative Medicine, Department of Medicine, Department of Basic Science, School of Medicine, Loma Linda University, Loma Linda, CA, USA.


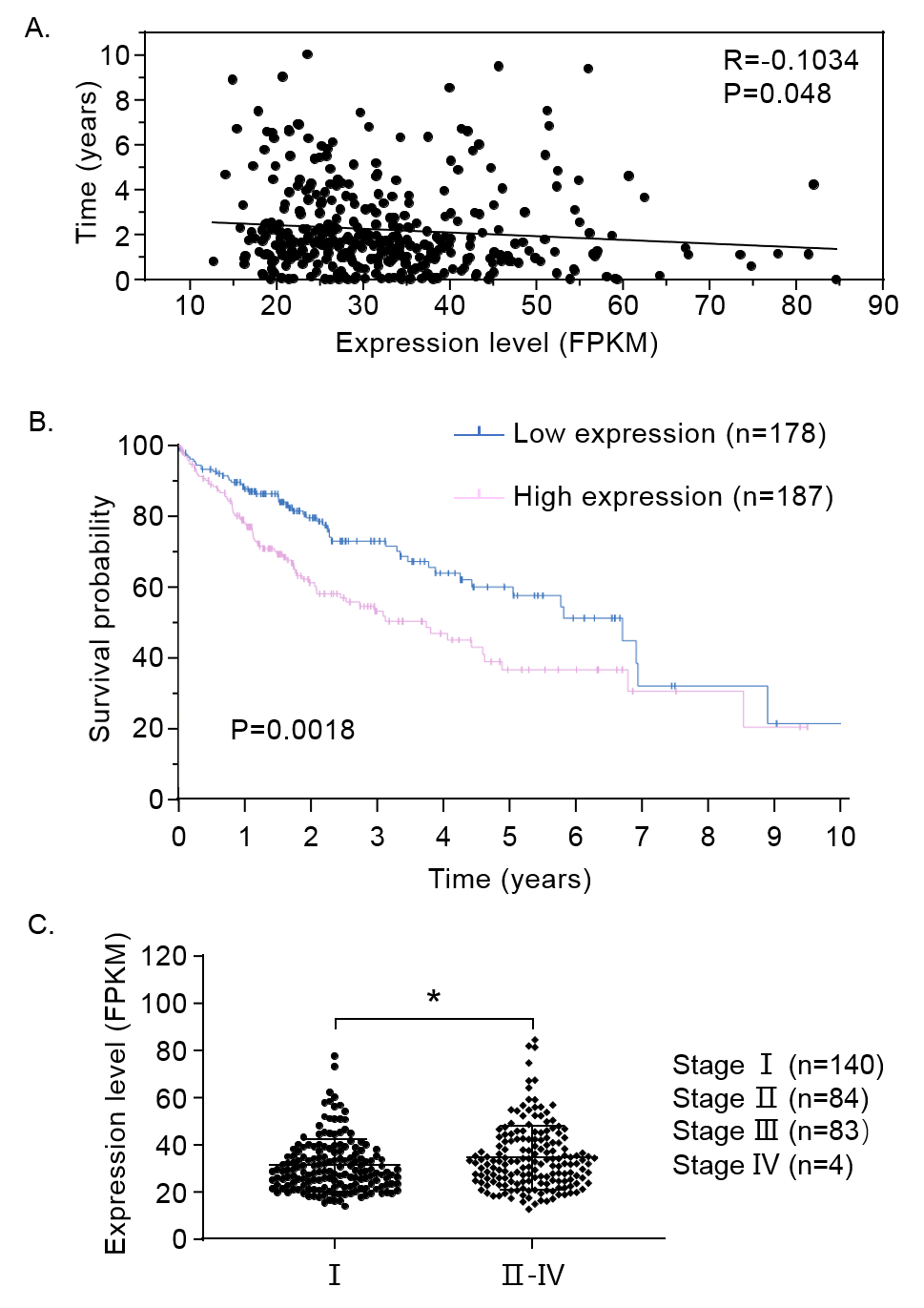


**Figure S1: NDUFB3 mRNA expression is inversely correlated with survival time and positively associated with disease stages. *A)*** Correlation between NDFUB3 mRNA expression levels and survival time. ***B)*** Survival probability of HCC with high and low NDFUB3 mRNA expressions. ***C)*** NDUFB3 mRNA expression levels between HCC stage I and advanced stages (stages II, III, and IV). **P* < 0.05.


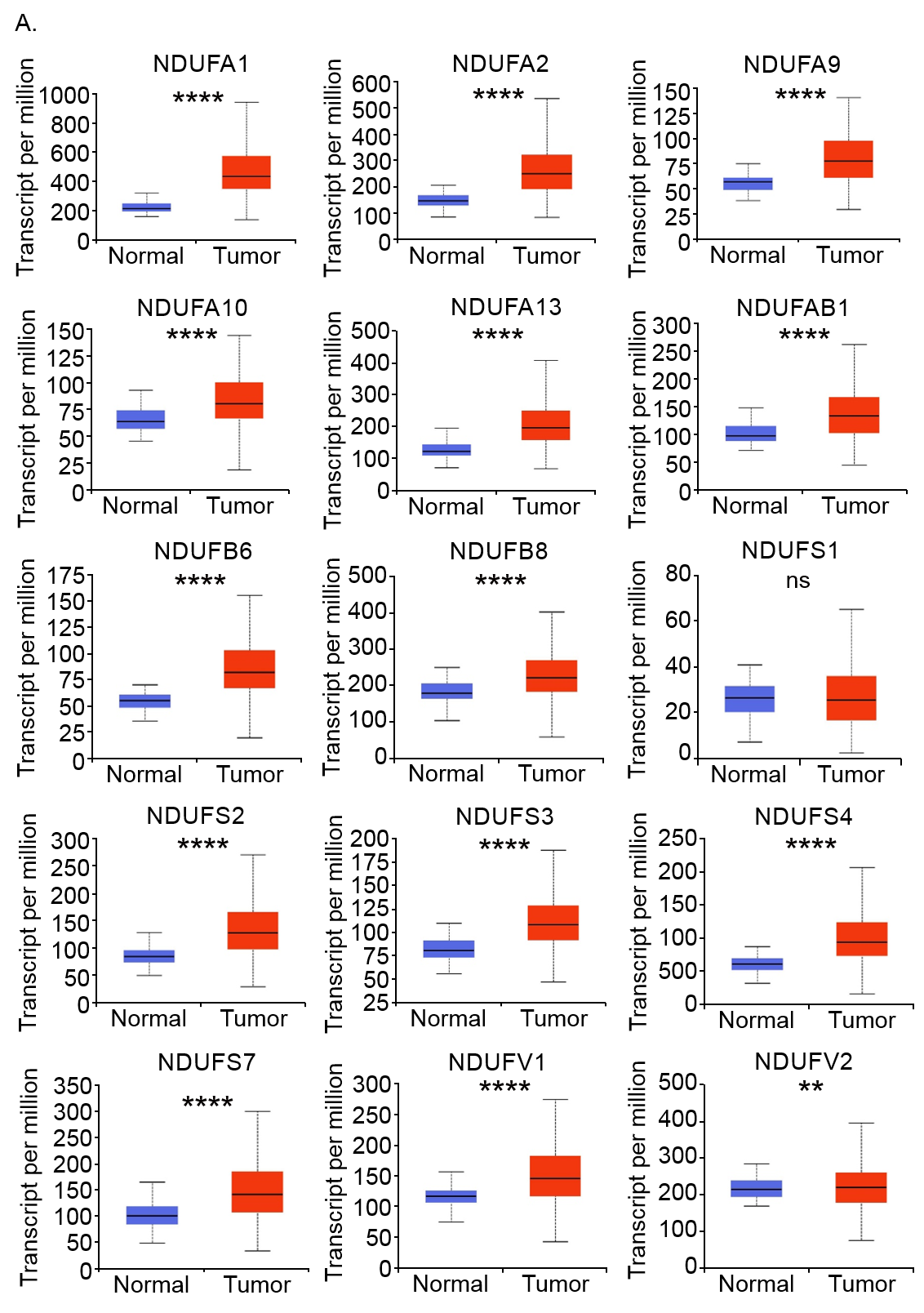


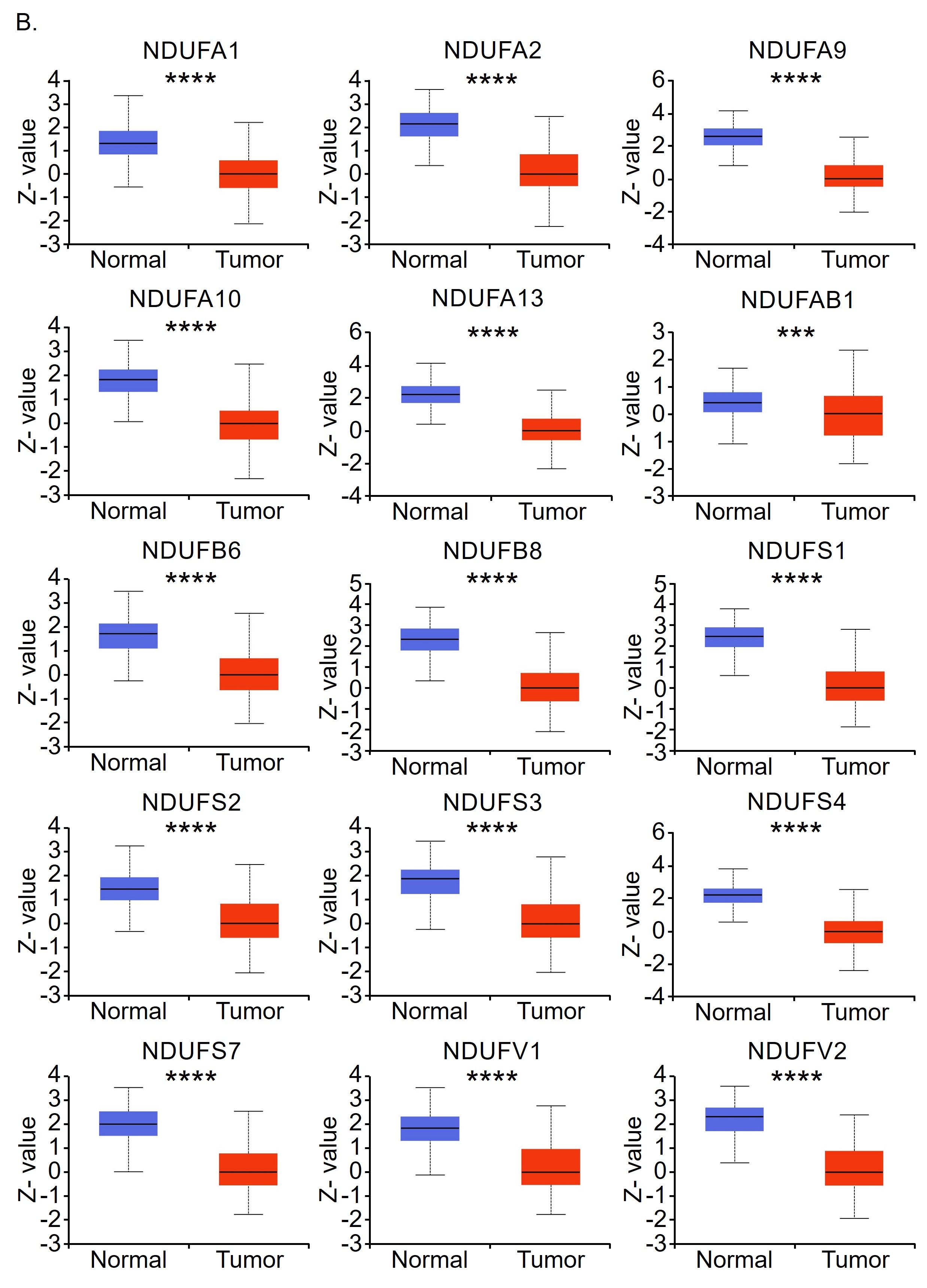


**Figure S2. The mRNA and protein expression levels of Complex I subunits in HCC (Tumor) and normal liver tissues (Normal)**. ***A)*** mRNA expression of the Complex I subunits in HCC and adjacent normal tissues in the TCGA database. ***B)*** Protein expression of the Complex I subunits in HCC and adjacent normal tissues was analyzed in the CPTAC database. ***P* < 0.01, ****P* < 0.001 and *****P* < 0.0001. “ns”: not significant.


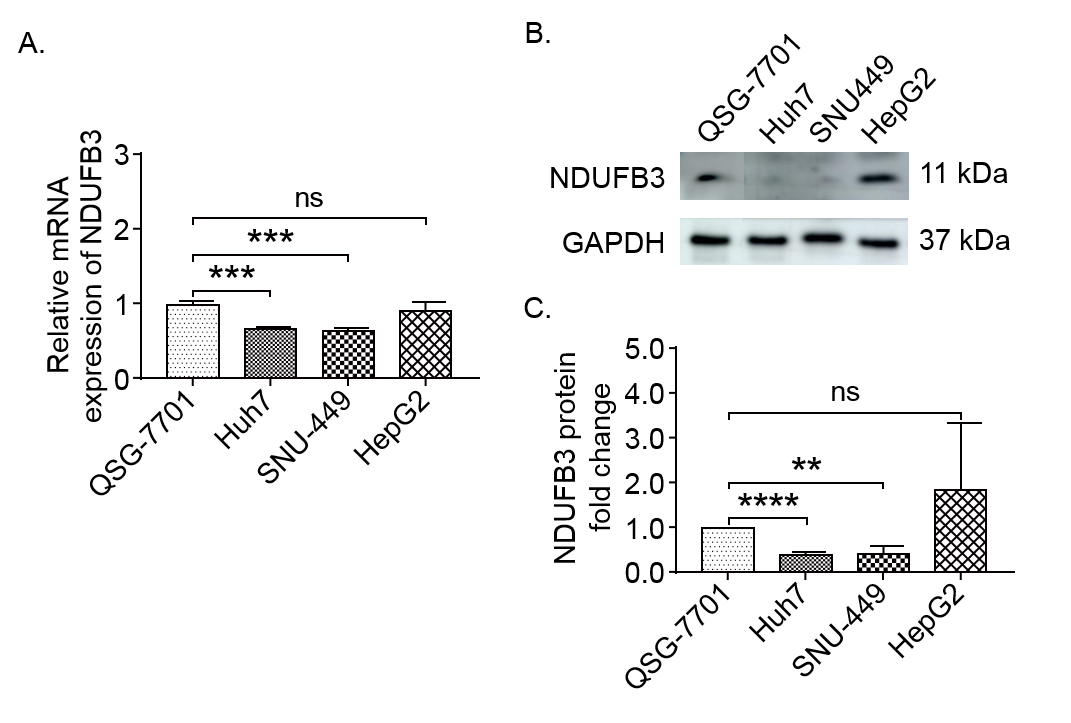


**Figure S3.** **NDUFB3 protein expression is decreased in some HCC cell lines.** ***A)*** RT-qPCR analysis of NDUFB3 mRNA expression in HCC cell lines (Huh7, SNU-449, and HepG2) and the normal hepatocyte (QSG-7701). ***B and C)*** Western Blot analysis of NDUFB3 expression in the HCC cell lines and the normal hepatocyte (QSG-7701). Representative gel images (B) and cumulative data (C) are shown. ***P* < 0.01, ****P* < 0.001 and *****P* < 0.0001. “ns”: not significant.


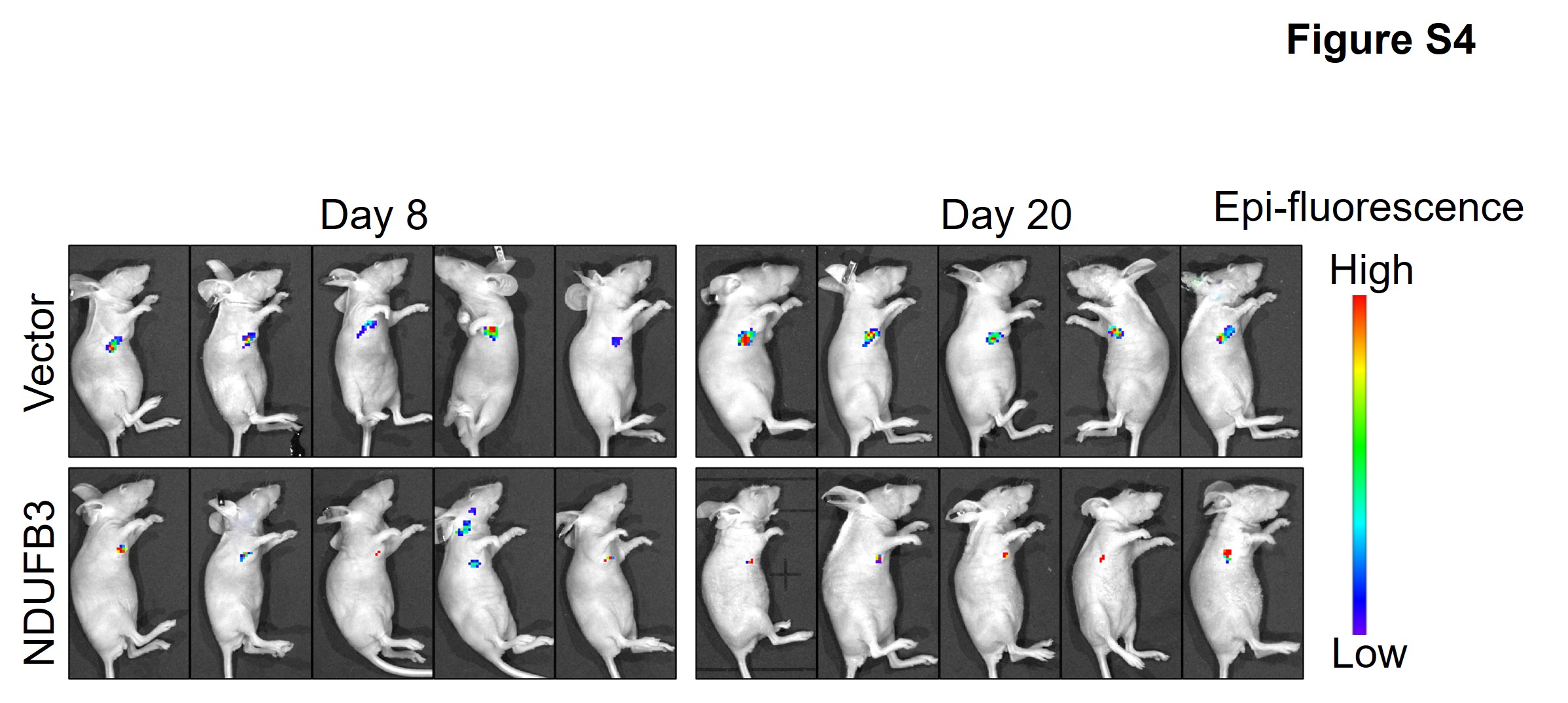


**Figure S4. *In vivo* growth of Huh7 cells in nude mice.** Female BALB/c nude mice were divided into two groups. 1 × 10^7^ control (Vector) or NDUFB3-overexpressing (NDUFB3) Huh7 cells in 100 μL were inoculated subcutaneously under the right axilla. The tumors were imaged in a small animal live imager on days 8 and 20 after the cell injection.


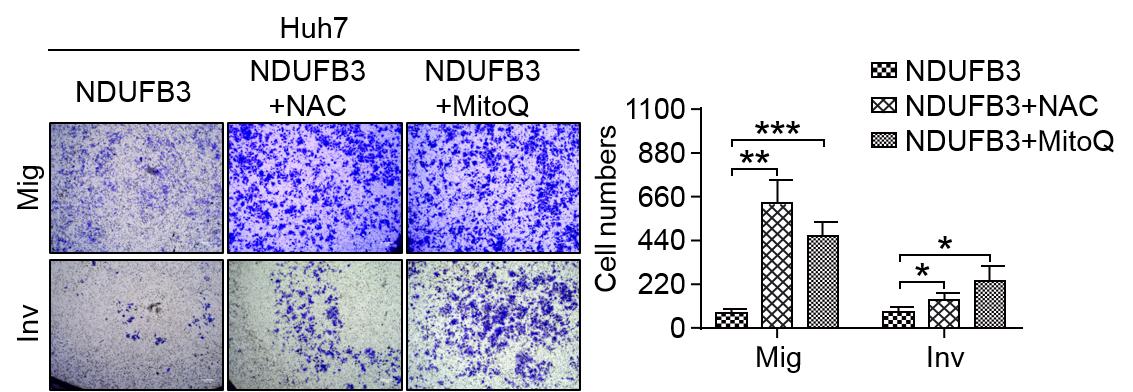


**Figure S5. Inhibition of cellular and mitochondrial ROS production reverses the effects of NDUFB3 overexpression on the migration and invasiveness of HCC cells.** NDUFB3-overexpressing (NDUFB3) Huh7 cells were examined for migration and invasiveness as described in Methods in the presence of one of the following treatments: ***a)*** none (NDUFB3), ***b)*** N-Acetyl cysteine **(**NAC, 10 nM; a cellular ROS scavenger) (NDUFB3+NAC), and ***c)*** mitoquinone mesylate (MitoQ, 5 μM; a mitochondrion-targeted ROS scavenger) (NDUFB3+MitoQ). Representative images (left panel) and cumulative data (right panel) are shown. **P* < 0.05, ***P* < 0.01, ****P* < 0.001.
